# The Equilibrium of Population and Sustenance Demonstrated; Shewing, on Physiological and Statistical Grounds, the Means of Obviating the Fears of the Late Mr. Malthus and His Followers

**Published:** 1836-04-01

**Authors:** 


The Equilibrium of Population and Sustenance demonstrated; shewing, on Physiological and Statistical Grounds, the Means of obviating the Fears of the late Mr. Malthus and his Followers. By *Charles Loudon,* M.D. Octavo. London, 1836.

When practically applied, Dr. Loudon's demonstration and his principle will operate powerfully in exposing some of the delusions of the Malthusian System; and, at the same time, they will exert a pure philanthropic influence in counteracting its inherent follies and falsities. According to the Doctor, there is no error in political economy so much calculated to retard the progress of civilization and welfare of the poor, as the idea that population is likely, by small allotments of land, to increase so much, that not only will the surface of the globe be covered with starving crowds of inhabitants, but that the greatest misery will prevail among mankind. Many visionaries have fancied that, when the earth is overspread with people, which they consider inevitable, men will be compelled to devour one another. Under this notion, and the dread of being unable to provide for offspring, certain doctrines have lately been promulgated, alike disgraceful to those who suggested them, and injurious to the interests of morality. Dr. L. therefore, proposes a check, both moral and healthful, which, if necessary, might be applied to population; and, as this check is considered to be based on physiology, and founded on the laws of Nature, we will bring it fully under the observation of medical readers, who, of all others, are the best qualified to appreciate such questions.

Dr. Loudon opens his essay by stating, that the increase of population, in these islands, is about ten per cent. every census of ten years; or, in other words, one per cent. annually: and that the periods of gestation and lactation, together, extend nearly to nineteen months. The term of gestation has been fixed by Nature at nine months—that of lactation is generally arranged by the habits of particular nations; on an average, ten months is the usual time in this and most European countries. Mothers generally do not become impregnated during lactation; but, after making allowance for exceptions, if the period of lactation be prolonged only a very few months, increasing with the increase of population, and *vice versâ,* the problem of the equilibrium of population and sustenance is solved—and on grounds which involve no disadvantage, either to mothers or their offspring. On the Continent, the average number of children to a marriage is nearly 4·5 ; in England it is the same. If, then, by prolonging the time of suckling, this average were reduced to four, it is evident that population would remain stationary, because one-half of our numbers dies between birth and the twenty-fourth year. With us, the average age of marrying is very near to that period (24 years) ; in France, it is twenty-six years—that of men being twenty-nine, and that of women being twenty-four. Now, if we adopt these numbers for both sexes in England, and thus admit the time of marriage, for females, to be twenty-four, and that of child-bearing to terminate at forty-four, the average interval between each child will be fifty-four months, or four years and a half. The next enquiry is—what increase of time, beyond the ten months of lactation, would be necessary to keep population in check, as it advances at present in England? The reply is—admit, in every instance, the nine months of gestation, the ten months of lactation, and one-tenth of the remaining thirty-five months as an equivalent for the present increase of population, and the period to which suckling should be extended will be *thirteen months and a half.* To this, however, must be added six weeks, for the chances of impregnation taking place during the three months and the six weeks, on the supposition that this occurs once in three instances of lactation. Hence, the Doctor concludes, the precise time will be fifteen months, or *one-third longer than the present number.* This extension of lactation, he adds, must necessarily increase the 4·5 years between each child approximating to 5, and, consequently, reduce the 4·5 children in a family to four : wherefore, since one-half of our numbers die under the age of marriage for females, the result will be—that there will remain only a representative for father and a representative for mother, on an average, in every family throughout the country.

With Dr. Loudon, we should like to know a physiological or other reason why our British mothers do not usually suckle their children longer than ten months. Certain it is, as the Doctor affirms, that the milk does not diminish particularly at that time, so far as regards the quantity ; and, from the health of children reared without spoon-meat beyond this time, as certainly it undergoes no change in its quality. Children are sometimes seen so old, before weaning, as to be able to ask for the breast; and it has not been remarked that the health of mothers, thus suckling, was in any way worse than that of their neighbours.* Altogether, then, it may be asserted, that a mother is likely to enjoy better health, and to be less liable to sickness and death during lactation, than during pregnancy. Many women believe, or affect to believe, that the weakness they labour under arises from some latent moral or physical cause ; but this weakness is not attributed to lactation in the earlier months of suckling, because the mother then considers herself fulfilling a necessary duty, which her constitution, for so long, is well able to bear. So soon, however, as the period of

---

* This review is written by a talented physician, of great experience and observation—and one of our oldest professional friends. We make this remark, in order that the Editors may not be considered as responsible for every opinion broached in this article. We would here observe, that we have great doubts whether the generality of British mothers in high life—and even among the commercial and professional classes of society, would bear lactation prolonged to the extent which Dr. Loudon advocates. We imagine that such a plan would prove a greater check than is supposed by either author or reviewer. We think it would deteriorate the health of a large proportion of females of the present day. There is one other consideration which may be adverted to. Would not the interval of four or five years between each child leave a large number young and unprovided for, at a period when the parents were disabled, by age, to see them set out in life ?—Eds.

lactation has passed over, as it is established by custom or fashion, she imagines she is exceeding the intentions of Nature, and she forthwith concludes that the continuance of suckling is the cause of her uncomfortable sensations. This whim being entertained, the child is weaned, and too often becomes the victim of a most reprehensible delusion.

Since Nature has furnished the mother with milk for a longer period than custom demands, it is evident that some good purpose for the mother and child, was intended in this arrangement. Had it been otherwise, the Doctor infers, the secretion of milk would stop at a definite time, in like manner as the period of gestation is definite. That a child, in comparison with the young of the lower animals, is so long unable to provide for itself, strongly tends to corroborate the proofs already advanced—that Nature originally had in view a more protracted period for lactation, than is now allowed. Some writers, following the laws of Nature, as they interpreted these, fixed the period of weaning at fifteen months, when the infant has got its eight incisors, and its four canine teeth. There are well authenticated instances of mothers having suckled their children for three, four, five, and even seven consecutive years: we ourselves have known cases of lactation being prolonged for three and for four years, with the happiest results.

Dr. Loudon continues the illustration of his doctrine by observing—that the period of lactation has a great influence over the numbers of mankind in various countries, as is evinced by numerous facts. He adduces proofs of this position. Thus, he says, in China, where the population is excessive, and the inhuman practice of infanticide is uncondemned, they wean a child as soon as it can put its hand to its mouth.* On the other hand, the Indians of North America do not wean their children until they are old and strong enough to run about: generally, they are suckled for a period of more than two years. Hence proceeds the thinness of these tribes in a fertile country, when compared with other barbarous and even civilized nations.† Among the Charribean races, the population is also remarkable for the paucity of numbers in connexion with a similar extent of location; and, what is of more importance still, such a number as seven or eight to a family is scarcely known. That a like cause exists for the decrease of the negro population in the West Indies, among both the Maroons and the *imported Africans*, is held by the Doctor to be highly probable. He considers this decrease as being more satisfactorily accounted for by protracted lactation, than by the cruelty so unjustifiably imputed to the planters by Mr. Fowell Buxton, with the herd of his insensate and garrulous abettors.‡

---

* Heretofore, as in modern days, when a people has advanced into a high state of civilization, it has ever happened that mothers have fallen insensibly into the guilty practice of renouncing altogether the duty of suckling their own offspring, or into the but little better habit of greatly shortening the term of lactation which, by fashionable matrons and their senseless imitators, is contemned as a vulgar encroachment on their pleasures and their ease. Hence it comes, that an overcrowded population usually accompanies the growing excesses of refinement and luxury.—*Rev.*

† The wandering life of the Indian, who subsists chiefly by the chace, is less favourable to increase of population than the life of the civilized settler, leading a life of agriculture, peace, and comfort. The state of constant warfare, too, in which the Indian tribes live, as well as the continued encroachments made on their hunting tracts by the Europeans, must be a most effectual check on population.—*Eds.*

‡ Surely Dr. Loudon and the reviewer must allow something for the life which a *slave* leads beneath the pestilent skies of the Antilles, even under the

While Dr. Loudon thus points out a moral and healthful check to the overgrowth of population, with the object of allaying the dread of prospective misery, he does not intend to inculcate the practical adoption of his principle, under the actual circumstances of our country. We, however, would advise a gradual and discriminate adoption of this important principle, among those families who are obliged to use the medical or pecuniary assistance of clubs and parishes; and, by encouraging such a course, their doctors would contribute greatly to the well-being of many industrious but improvident operatives, and also assist essentially in promoting the best interests of society.*

Those persons who have considered the relations of population to sustenance in America, Dr. Loudon further observes, must clearly see that the resources of mankind for the production of food, in the western world alone, are such as to meet every possible increase of population, for an indefinite number of ages to come. We cannot conceive to what extent the population, even of this country, may reach, without incurring the apprehension of a scarcity of food, as it is impossible to foresee the extent of the improvements which the progress of the arts and sciences will effect in agriculture.

With regard to those products which are strictly vegetable, the beet-root modern cabbages, potatoes, and Swedish turnips, are instances of immense crops arising from a small surface of ground. The improvements by drainage, spade-husbandry, and manures of various kinds, have greatly increased the quantity of grain, while the method of open and close stall-feeding will contribute exceedingly to the store of animal food. Besides, the extensive means of conveying fresh fish inland from the coast, and indeed every thing necessary for man, as well as agriculture, by the rail-roads now constructing, will speedily make important changes even in those districts where improvements were considered as next to impossible. Thus, in a few concise notes, Dr. L. has suggested a diversity of subjects which, in all their bearings, ought to occupy a prominent place among the themes of medical investigation. Throughout his Essay, the Doctor maintains the bright character of an enlightened philanthropist, distinguished alike for his intelligence and sagacity.

Dr. Loudon has ascertained, that there are seventy-six millions and a half of acres of land in the United Kingdom, and thirty millions of these are waste; and, that one half of these thirty millions is capable of improvement. He finds, that each arable acre, yielding an average crop of potatoes, will produce thirty tons, or sixty-seven thousand two hundred pounds, of potatoes annually; which, being divided by the three hundred and sixty-five days of a year, will give one pound for each of four meals, or four pounds a-day, during the whole year, for forty-two persons. Hence, it is manifest that two millions and a half of acres of potatoes will permanently produce vegetable food for upwards of one hundred millions of people, or four times our present population; allowing the remaining seventy-four millions of acres to produce animal food, grain, and the other commodities of sustenance, exclusive of what may be imported from our colonies and foreign countries. The Doctor abstains, as irrelative to his inquiry, from

---

most kind-hearted master! But, even with all disadvantages, it is well known that the African population, especially in the southern states of America, increases considerably.—*Eds.*

* With this sentiment we entirely agree. Indeed every medical observer must be convinced that a considerable proportion of mothers in the better classes of society, wean their infants, or put them out to nurse, more for the indulgence of their own pleasures, than from want of health. It is thus the sons and daughters of our aristocracy are obliged to be quartered on the nation, in the same way that the offspring of paupers are thrown on the parish! So true is it, that extremes meet.—*Eds.*

pointing out the alternations and changes of the various crops, or how far it might be practicable to bring into cultivation even a large portion of the fifteen millions of acres represented as incapable of improvement. He refers to the authors who have written on the subject of sterile fields, and to the historical records connected with the immense populations of Egypt, Palestine, Greece, and Italy, in ancient times, as being calculated to furnish some idea of what can be done with lands of the most unpromising nature. He limits and directs our attention chiefly to two facts having intimate relations with medicine: one of these is physiological, the other dietetic: and, both of them exercise an incalculable influence on the happiness and welfare of every order of the state. The physiological principle of lactation, as effecting the increase or decrease of population, has never yet been demonstrated statistically by any previous writer on population or physiology. Dr. Loudon is the first to open the culture of this extensive field, and we would entreat of him to prosecute his investigations. The dietetic principle has hitherto been but little attended to, on national grounds; and the Doctor ascribes such neglect to a lurking apprehension, on the part of legislators, that there are no rational means appointed by Nature for suspending the advance of population, or of promoting its increase, in geometrical proportions. We invite our readers, in their professional intercourse with all grades of society, to disseminate the knowledge of these two fundamental principles, and their momentous influences on the power, the peace, and the prosperity of nations.

---

